# African coastal camera network efforts at monitoring ocean, climate, and human impacts

**DOI:** 10.1038/s41598-023-28815-6

**Published:** 2023-01-27

**Authors:** G. O. Abessolo, R. Almar, D. B. Angnuureng, F. Bonou, Z. Sohou, I. Camara, A. Diouf, G. Alory, R. Onguéné, A. C. Mama, C. O. T. Cissé, B. A. Sy, I. Sakho, S. Djakouré, S. Yao, A. R. Tano, E. W. J. Bergsma, O. A. Dada

**Affiliations:** 1 Ecosystems and Fishery Resources Laboratory, Institute of Fisheries and Aquatic Sciences, University of Douala, BP 2701 Douala, Cameroon; 2 LEGOS, OMP, UMR 5566 (CNES-CNRS-IRD-University of Toulouse), Toulouse, France; 3 Africa Centre of Excellence in Coastal Resilience, CCM, University of Cape Coast, Cape Coast, Ghana; 4 Laboratoire d’Hydrologie Marine et Côtière, Institut de Recherches Halieutiques et Océanologiques du Bénin (IRHOB)/Laboratoire de Physiques et Applications, LPA/Université Nationale des Sciences Technologies, Ingénierie et Mathématiques (UNSTIM), Abomey, Benin; 5 Chaire Internationale de Physique, Mathématiques et Applications, Cotonou, Benin; 6 Laboratoire d’Hydrologie Marine et Côtière, Institut de Recherche Halieutique et Océanologique du Benin (LHMC-IRHOB), Cotonou, Benin; 7 Cheikh Anta Diop University, Dakar, Senegal; 8 Laboratoire d’Océanographie, des Sciences de l’Environnement et de Climat (LOSEC), Université Assane Seck, BP 523, Ziguinchor, Sénégal; 9 Technology and Applied Science Laboratory, University Institute of Technology, University of Douala, P.O. Box 8698, Douala, Cameroon; 10 Laboratory Leïdi “Dynamics of the Territories and Development”, University Gaston Berger, BP 234, Saint-Louis, Senegal; 11 Technologies Avancées et Développement Durable, Université Amadou Mahtar Mbow de Dakar a Diamnadio, UMR Sciences, BP 45927, Dakar, Senegal; 12 Univ Rouen Normandie, UNICAEN, CNRS, M2C UMR 6143, 76000 Rouen, France; 13 Laboratoire des Sciences de la Matière, de l’Environnement et de l’Energie Solaire (LASMES)/UFR SSMT/Université Félix Houphouët-Boigny, Cocody, Côte d’Ivoire; 14 Laboratoire de Physique et de Géologie Marine, Centre de Recherches Océanologiques, Abidjan, Côte d’Ivoire; 15 Laboratoire de Physique Fondamentale et Appliquée, Université NANGUI ABROGOUA, Abidjan, Côte d’Ivoire; 16 CNES, 18 Avenue Edouard Bélin, 31400 Toulouse, France; 17 Department of Marine Science and Technology, Federal University of Technology, Akure, Nigeria

**Keywords:** Physical oceanography, Natural hazards, Climate change, Environmental impact, Geomorphology

## Abstract

Regular and long-term monitoring of coastal areas is a prerequisite to avoiding or mitigating the impacts of climate and human-driven hazards. In Africa, where populations and infrastructures are particularly exposed to risk, there is an urgent need to establish coastal monitoring, as observations are generally scarce. Measurement campaigns and very high-resolution satellite imagery are costly, while freely available satellite observations have temporal and spatial resolutions that are not suited to capture the event scale. To address the gap, a network of low-cost, multi-variable, shore-based video camera systems has been installed along the African coasts. Here, we present this network and its principle of sharing data, methods, and results obtained, building toward the implementation of a common integrated coastal management policy between countries. Further, we list new contributions to the understanding of still poorly documented African beaches’ evolution, waves, and sea level impacts. This network is a solid platform for the development of inter-disciplinary observations for resources and ecology (such as fisheries, and sargassum landing), erosion and flooding, early warning systems during extreme events, and science-based coastal infrastructure management for sustainable future coasts.

## Introduction

Coastal areas contain some of the world’s most diverse and productive resources that support a variety of economic activities, including fisheries, tourism, recreation, and transportation. They are, nevertheless, extremely vulnerable to human intervention and climate change. Their recreational, ecosystem and protection services and economic values give them high societal attention, especially given that these areas are home to more than a trillion dollars' worth of infrastructure and investments globally^1,2^. Many coastal areas have undergone extensive modification and development over the past few decades, greatly increasing their vulnerability to both the anticipated effects of global climate change and the natural coastal dynamics^3,4^.

**Figure 1:**
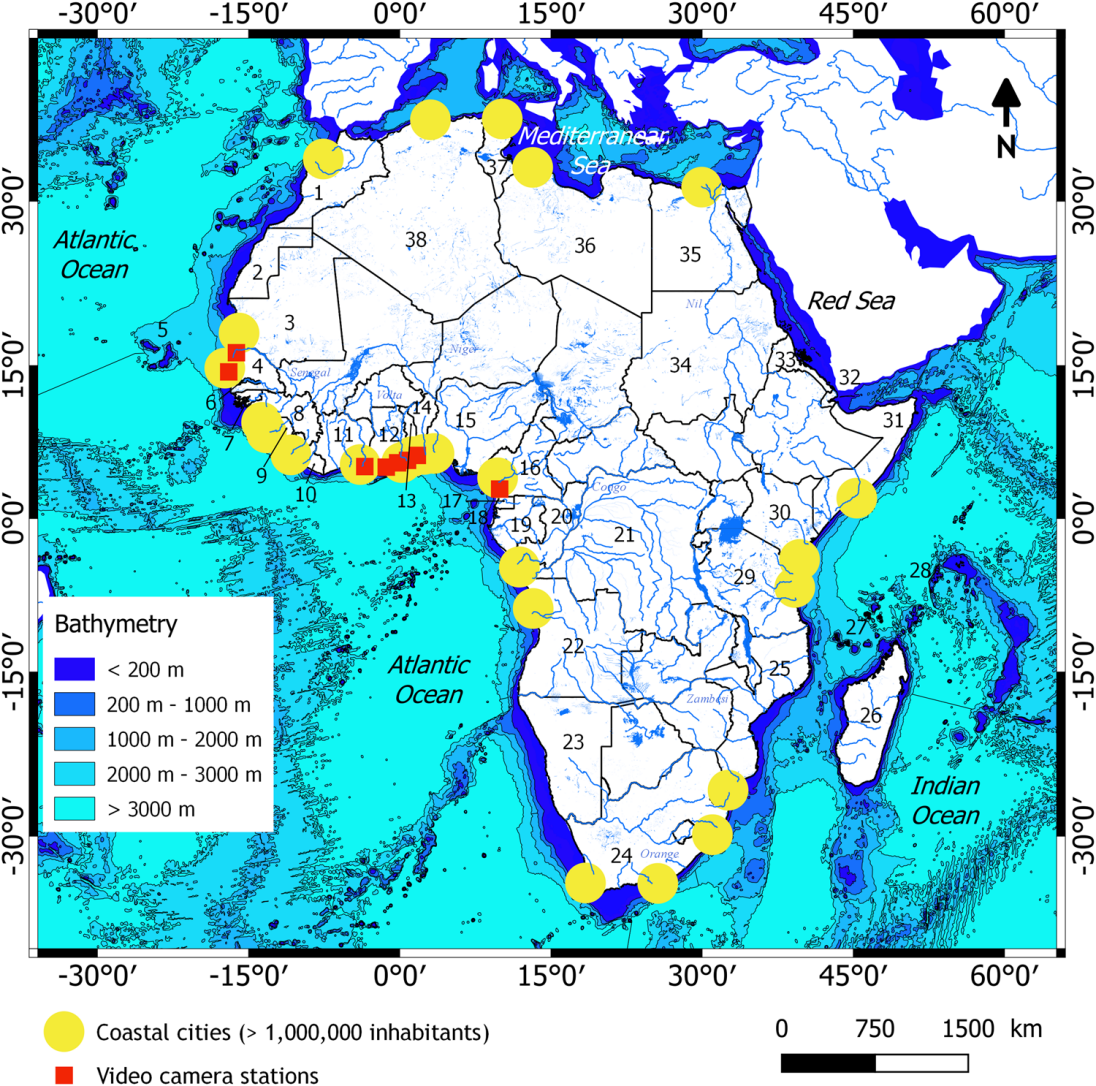
African coastal countries with inland waters and rivers: Morocco (1), Western Sahara (2), Mauritania (3), Senegal (4), Cape Verde (5), Gambia (6), Bissau Guinea (7), Guinea (8), Sierra Leone (9), Liberia (10), Côte d’Ivoire (11), Ghana (12), Togo (13), Benin (14), Nigeria (15), Cameroon (16), Equatorial Guinea (17), Sao Tome-et-Principe (18), Gabon (19), Republic of the Congo (20), Democratic Republic of the Congo (21), Angola (22), Namibia (23), South Africa (24), Mozambique (25), Madagascar (26), Comores (27), Seychelles (28), Tanzania (29), Kenya (30), Somalia (31), Djibouti (32), Eritrea (33), Soudan (34), Egypt (35), Libya (36), Tunisia (37), Algeria (38). In red, the installation sites of the video camera systems, which are: Saint Louis (Senegal), Mbour (Senegal), Assouindé (Côte d’Ivoire), Elmina (Ghana), James Town (Ghana), Dzita (Ghana), Grand Popo (Benin), Kribi (Cameroon). See Fig. 2 for more details on these sites. Bathymetry was derived and downloaded from the GEBCO website. Rivers’ data were downloaded from www.naturalearthdata.com. The figure was generated using *QGIS 1.18.15* software.

Africa's coastal zone extends over 41,184 km, covering 32 continental countries. Most of the continent's economic activities are located in coastal areas, with the presence of industrial zones around the main deep-water harbours. This has led in recent decades to a demographic explosion, and major and rapidly expanding cities on the coast. Indeed, each year, 10 million people are added to the urban population of sub-Saharan Africa^5^. Accordingly, several cities along the African coasts have more than one million inhabitants (see Fig. 1). The African coasts are subject to other activities such as artisanal fishing (e.g.,^6^), logging for firewood and charcoal (e.g.,^7,8^), sand mining (e.g.,^9^), tourism (e.g.,^10,11^), as well as onshore and offshore industrial activities (*e.g.*, processing industries, offshore oil extraction). Such activities lead to pressures and conflicts over the use of resources, often resulting in environmental degradation, which can threaten their development potential (e.g.,^12–14^). Small-scale fishing towns and natural ecosystems are often the first victims of the induced vulnerability, as they are highly sensitive to climate variability and change due to their low adaptive capacities, which are insufficient to cope with future climate change^15^.

Africa is the continent with the highest fraction of sandy beaches and low-lying coastal areas^16,17^ and is therefore very exposed to ocean, climate and human activities impacts. Indeed, several recent works showed that in recent decades, the coastal environment has been facing several disturbances in wave forcing and shoreline stability in West Africa (e.g.^18–22^), in North Africa (e.g.^23,24^), in Central Africa (e.g.^25,26^), in Southern Africa (e.g.^27,28^) and in East Africa (e.g.^29^). These works highlight that as a result, the Africans coasts have become particularly vulnerable to erosion and flooding due to high-energy storm waves, extreme storm-surge water levels, sea level rise, and the high proportion of sandy low-lying coastlines. In West Africa, this vulnerability is particularly aggravated by the decrease in rainfall and the presence of dams on the main rivers (*Volta* and *Niger Rivers*) thus reducing the supply of sediments to the coast^30^. According to^17^, 20% of the 284 reported African coastal natural and cultural heritage sites are currently at risk from an extreme coastal event, including the iconic ruins of Tipasa (Algeria) and the North Sinai Archaeological Sites area (Egypt). The vulnerable sites are expected to triple by 2050 if greenhouse gas emissions are high^17^.

Decision-making for coastal management in Africa remains a challenge in the face of increasing risks to people and their property^12,17^, as several factors impede the sustainable development of these areas. Firstly, multi-scale morphological evolution remains poorly understood on tropical coasts^31^. To ensure more integrated and efficient management of coastal areas, it is important to characterize the natural coastal systems, the dominant processes, and their integrated effects adequately. Secondly, there are few local indicators to predict the large-scale (spatial and temporal) impact of coastal infrastructure construction and development, and changes in ocean forcing on coastal morphology^23,32^. Numerical modelling would undoubtedly enable the derivation of these types of indicators^17,33,34^. Nevertheless, calibration and improving existing models require large databases and better descriptions of nearshore processes. Thirdly, the discordance of actors and responsibilities among African countries, and sometimes even within the same country, have too often prevented the planning of effective and sustainable long-term solutions^34^. There are regional programs that synergize efforts to understand the dynamics of these environments such as the West African Coastal Areas (*WACA*) Management Program and the West African Coastal Observatory (*ORLOA*). But the results of these integrated programs and their impacts are not yet prominent.

Effective decision-making for coastal management in Africa, therefore, requires the best and most up-to-date science. And this depends on the ability to collect long-maintained and real-time data, necessary to interpret and predict nearshore processes occurring at multiple temporal scales. Pioneering field measurement campaigns have been conducted for this purpose, providing crucial baseline measurements of short-term time-scales processes (e.g.,^31,35^). However, they are expensive and limited to timescales of days to weeks. The use of satellite radar and optical images enabled large spatial coverage of morphological variations. It is, however, limited to resolutions of tens of meters (10 m for Sentinel, 30 m for LandSat) with repeatability of several days (2–5 days for Sentinel 2^36^), thus limiting the long-term observation of short-term processes. Moreover, altimetry, which measures sea surface variations, is still limited to areas a few km from the coast because of inappropriate sensors for the nearshore. While satellite earth observation represents a real opportunity to monitor African coasts^37^, they face several challenges, including the lack of ground truth observations. Buoys allow the measurement of hydrodynamic parameters (e.g., waves, sea level, and currents) closest to the coast, but they are almost non-existent or unavailable along the African coasts. Purchase and installation of buoys are, however, expensive, and their maintenance is very complex, especially for a network covering large regions. And African coasts face a lack of historical data from tide gauges limiting the knowledge of climate-induced changes^38^.

Nearshore video camera systems are a low-cost option for high-frequency continuous long-term monitoring, with the advantage of being complementary to traditional measurement techniques (satellite, buoys, tide gauges, etc.) and particularly suited for tropical developing countries and open-wave areas^31^. Several parameters are estimated from video imagery, such as the shoreline position (e.g.,^39,40^), intertidal topography (e.g.,^41^), nearshore bathymetry (e.g.,^42–44^), breaking wave height (e.g.,^39^), sea level variations (e.g.,^45,46^), nearshore currents (e.g.,^47^). Video cameras allow for the collection of hard-to-access data on a continuous, long-term basis, effectively filling the data gap and, most importantly, ensuring effective monitoring of ocean conditions and beach changes^2^.

**Figure 2:**
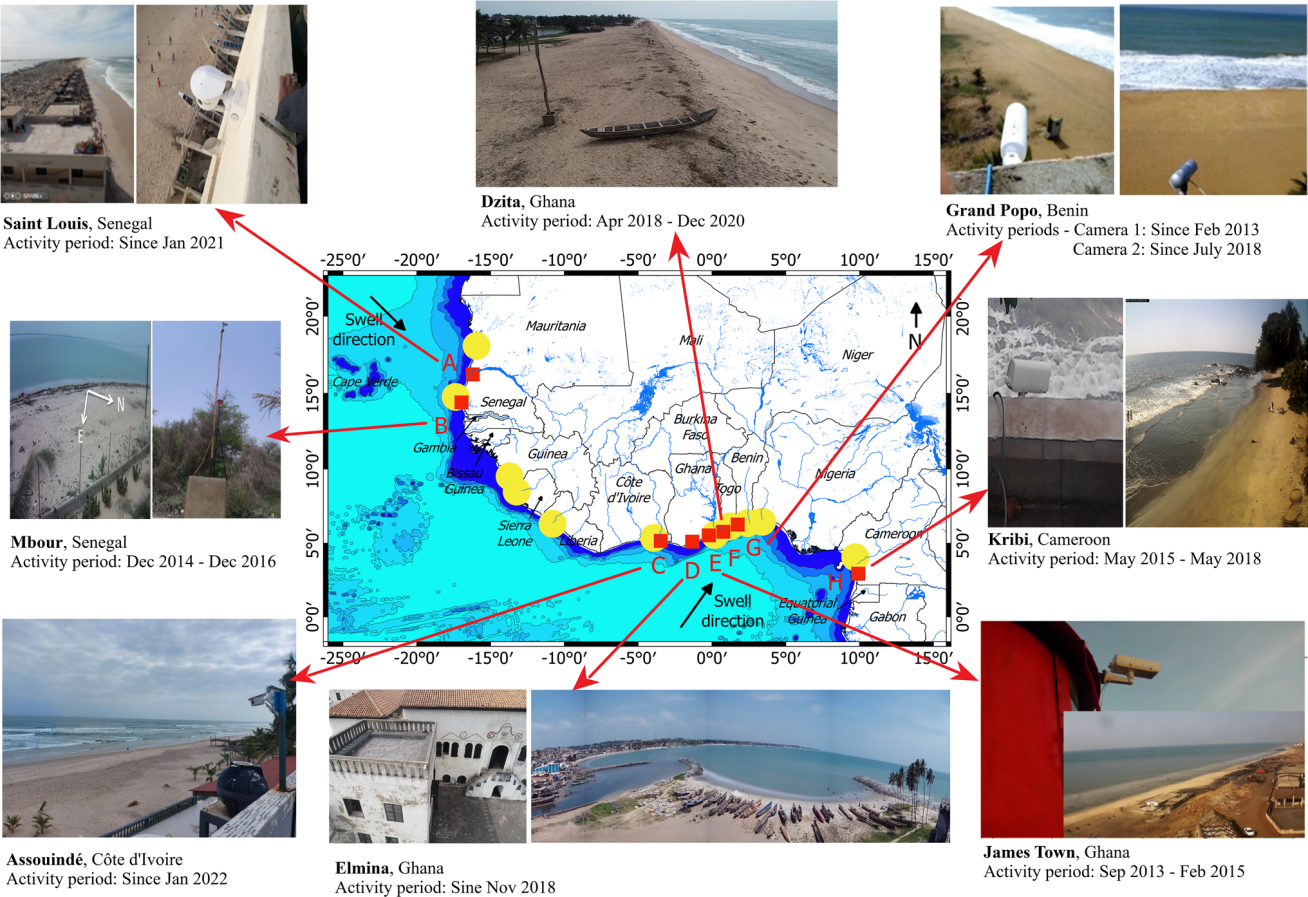
The African coastal camera network. The photos taken on the field show the environment of the installation sites and the installed cameras. The figure was generated using *Paint 3D* available in *Windows 10 Professional* from *Microsoft Corporation*. See Table S1 for technical, morphological, and hydrodynamic details of each video camera system.

A pilot video camera system was installed at Grand Popo (Benin) in 2013^31^, and the network is currently being extended to West and Central Africa (Fig. 2). In this paper, we demonstrate how this pilot network can be used to build a database to document African coasts' evolution, and how the network can support effective land-use planning policy. We present the main scientific advances resulting from the data collected, and we discuss the benefit of building data and scientific expertise in the management and planning of the coastal environment in Africa.

## New insights through coastal video camera monitoring

### All-in-one measuring system at the coast: methodological developments

**Figure 3:**
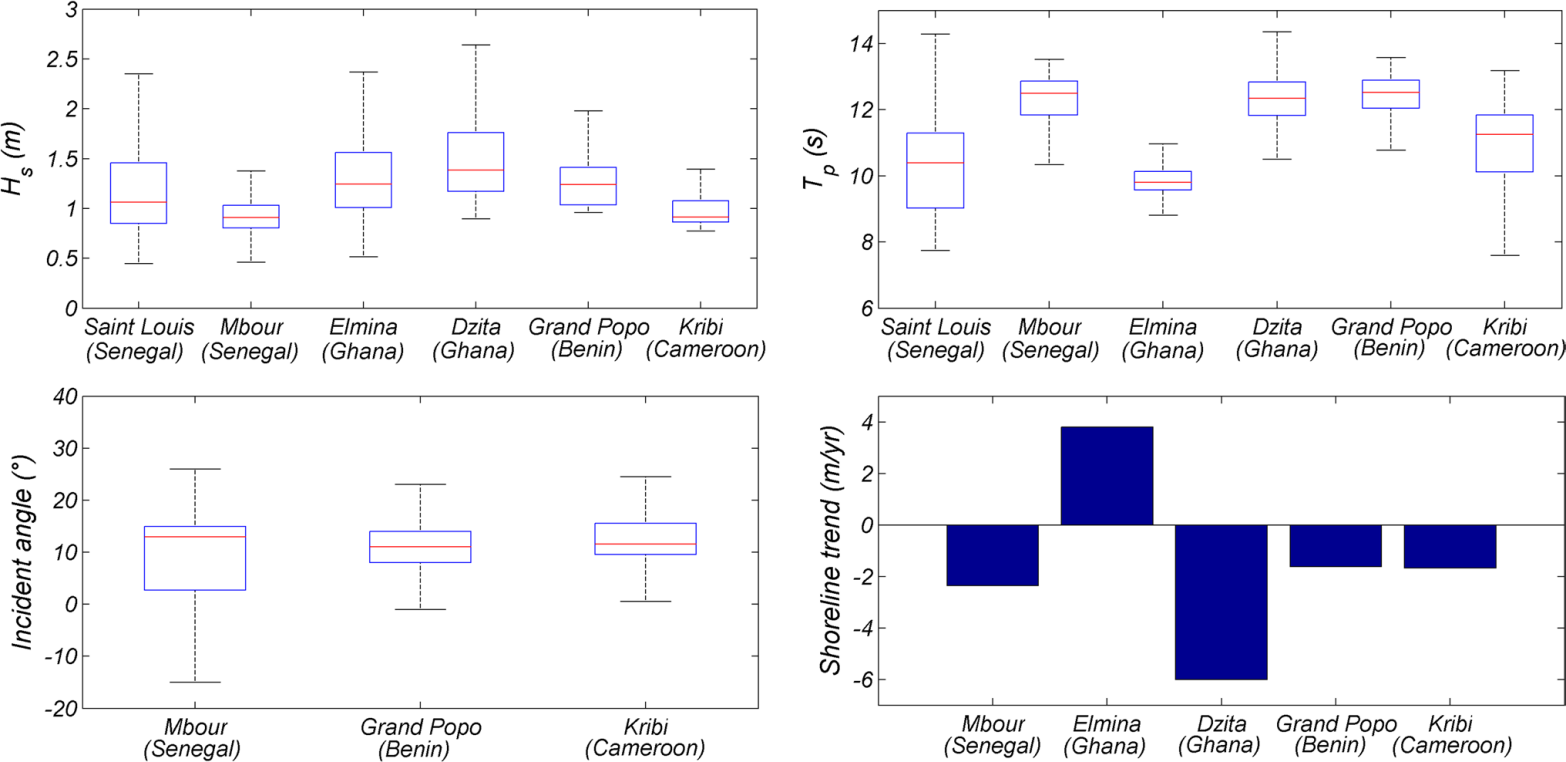
Wave characteristics (significant wave height *H*_*s*_, peak period *T*_*p*_ and incident angle to shore) and interannual shoreline trend derived from the African coastal camera network. The figure was generated using *MATLAB R2013a (8.1.0.604)* software.

Most of the parameters needed to understand the African coastal environments are measured simultaneously in this network: wave characteristics, water level variations and beach morphology. Figure 3 shows wave characteristics and interannual shoreline trends obtained from this network. Sites do not have the same estimated variables due to differences in local environments and existing coastal infrastructures and defenses. Several studies have been conducted to investigate these video-based estimates and the results showed in general an overall error below 10% (see results in Table S2).

**Figure 4:**
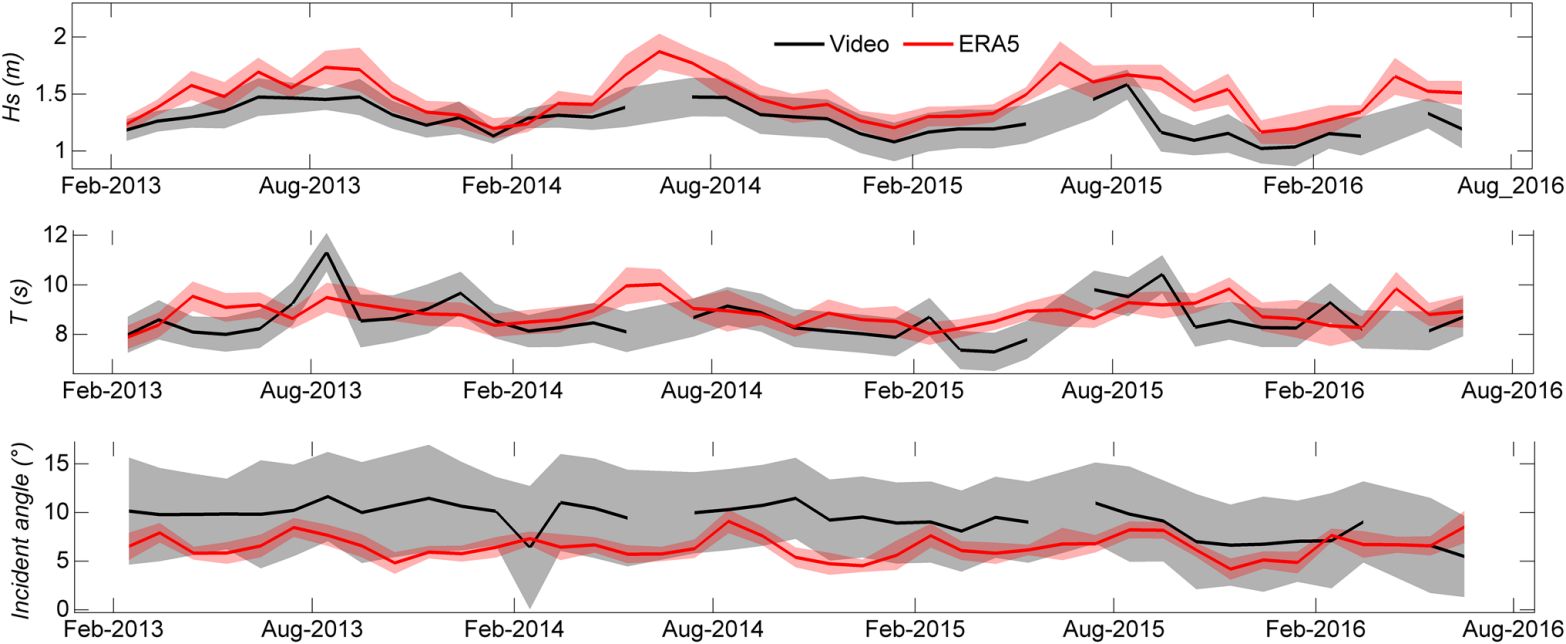
Comparison of video-derived wave estimates (black) with European Re-Analysis (ERA5) data (red) at Grand Popo (Benin) at monthly scale: significant wave height (*H*_*s*_), period (*T*) and incident angle. ERA 5 is a global atmospheric reanalysis developed by the Copernicus Climate Change (C3S) and produced at the European Centre for Medium-Range Weather Forecasts (ECMWF). ERA 5 data are freely available at www.ecmwf.int/en/forecasts/datasets/reanalysis-datasets/era5. ERA5 waves were propagated to the shore using the formula by^51^. Shaded areas indicate the standard deviation of all days in the month. The figure was generated using *MATLAB R2013a (8.1.0.604)* software.

The main characteristics of waves (e.g., Figs. 3 and 4) that represent the most important forcing in open coastal environments are determined in the nearshore where it is difficult to install conventional measuring equipment due to harsh and energetic wave conditions. Using in-situ measurements^48^, showed that wave dynamics are well captured by video camera systems (see Table S2 for errors). Video camera system provides long-term local wave datasets^49,50^ with much better accuracy than regional coarse available data (e.g. global coarse European Re-Analysis ERA5—Fig. 4). Available models are very often inaccurate when they are simplified for fast computational needs (e.g.,^51^), or require a lot of computational power, often out of the reach of African countries, to account for complex coastal processes and wave transformation.

**Figure 5:**
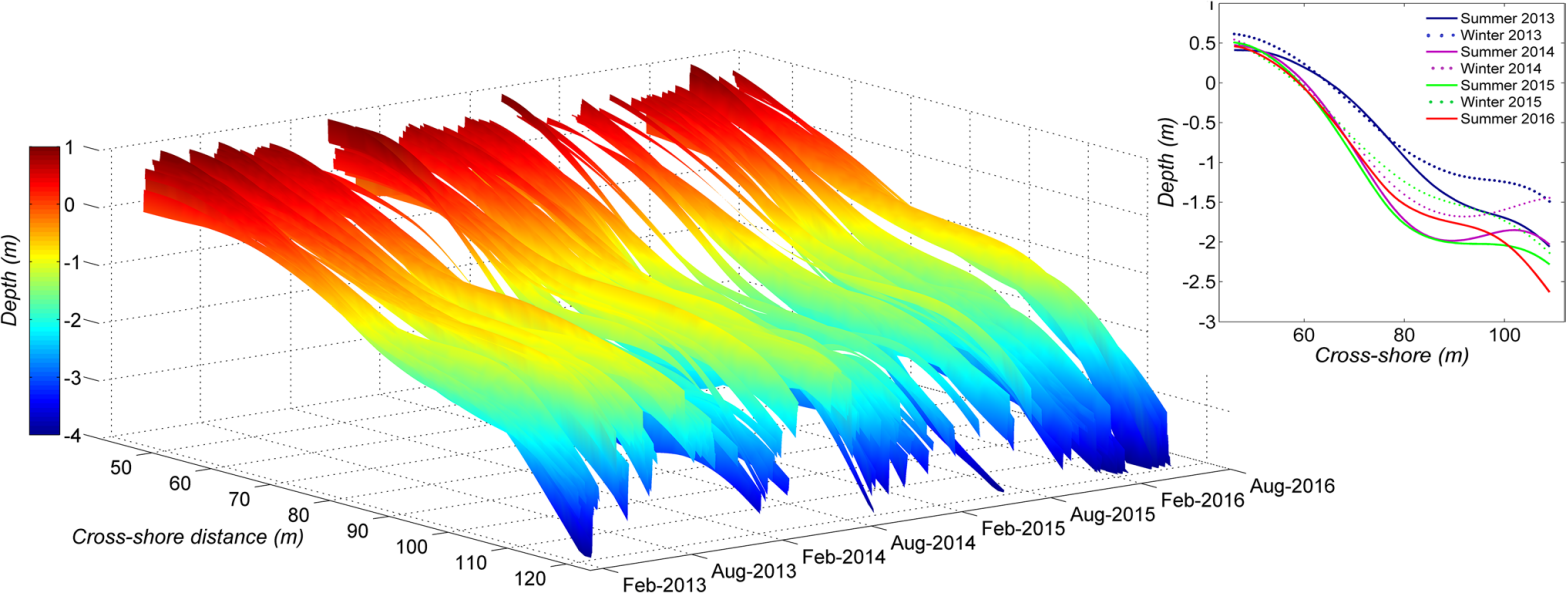
Combined intertidal topography and bathymetry profile obtained by processing data from the video camera system installed in Grand Popo, Benin, between February 2013 and August 2016. The upper rigth panel shows the average profiles over the seasons/years. The figure was generated using *MATLAB R2013a (8.1.0.604)* software.

In addition, monitoring morphological variations of a beach profile is shown to be feasible (Fig. 5) on a daily scale and over years from a video camera system^44^. The accuracy of topo-bathymetric measurements is crucial, as they are probably the most critical variables useful for understanding and modelling coastal dynamics and variability^42^. However, benchmark data are not always available to assess the accuracy of video estimates. A fitting error has been implemented in the spectral method cBathy^42^ to identify the erroneous data, and extract a set of useful data using a Kalman filter. For the temporal method^43,46^, defined two new error estimators based solely on video processing to assess the accuracy of video-derived bathymetry: the wavelength-related-error proxy and the tide-related-error proxy. These proxies express a reliability rate on bathymetric estimates to filter erroneous data when applying a Kalman filter^52^ tested these two proxies on data collected at Grand Popo, Benin, and showed that both estimators reduce errors by at least 30%.

### Capturing coastal water level

**Figure 6:**
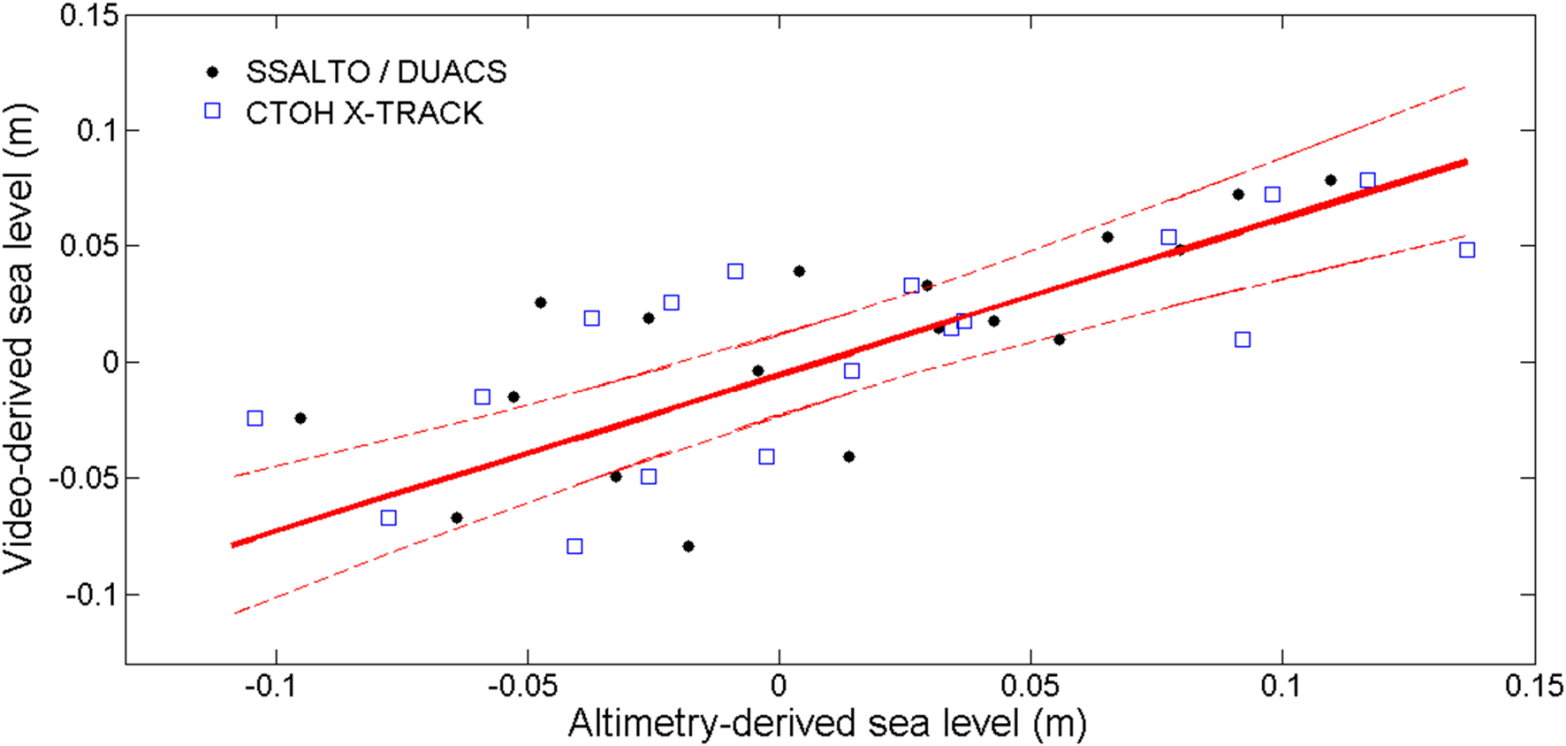
Comparison of monthly video-derived sea level anomalies with SSALTO/DUACS (black), and CTOH X-TRACK 2017 (blue) monthly derived sea level anomalies at Grand Popo, Benin. Red solid line are linear regression and red dashed lines indicate the 95% confidence levels. CTOH X-TRACK 2017 are altimetry products obtained from along-track combined TOPEX/Poseidon, Jason-1, Jason-2, and Jason-3 missions, at a spatial resolution of 6–7 km^84^. CTOH X-TRACK products (https://doi.org/10.6096/CTOH_X-TRACK_2017_02) are provided by the "Center for Topographic Studies of the Ocean and Hydrosphere (CTOH/LEGOS). The SSALTO/DUACS (https://doi.org/10.24400/527896/a01-2022.011 version 1.0) are SSALTO/Data Unification and Altimeter Combination System (DUACS) multi-mission gridded and delayed-time altimetry products provided by Copernicus Marine and Environment Monitoring Service. In the SSALTO/DUACS products, available altimeter missions are merged and mapped daily onto a 1/4°-resolution grid. See Table S2 for statistical values. The figure was generated using *MATLAB R2013a (8.1.0.604)* software.

There is an obvious need to extend the satellite-based sea level record toward the coast, where altimetry cannot yet reach, and where tide gauges are difficult to install. Figure 6 shows that the video-derived sea level measurements are in good agreement with two existing coastal altimetry products used for comparison^45^. Tidal harmonics were successfully extracted together with long-term sea level rise. And the signature of sea level variations induced by coastal waves, such as Kelvin waves, could well be present in the video data, but this remains to be investigated through more developments. Video stations help in understanding the main factors that determine the accuracy of near-shore altimeter data; and the propagation of sea level variations to the coast (see^53^). In addition, imagery from video stations is needed to refine estimates of wave contribution to sea level variations at the coast, which have so far been estimated by empirical formulations^54^. Indeed, waves undergo a series of complex transformations when propagating from deep-water to shallow water, resulting in the well-known process of wave set-up^55^ which adds a physical contribution to sea level variations. Using a collocated dataset, tide gauge, and wave buoy, Abessolo et al.^53^ showed that wave set-up contribution could be estimated from a video camera system, thus highlighting the potential of optimizing the location of coastal observation networks with respect to satellite ground tracks (as expressed in^56^). A new method for runup detection from video imagery^57^, consisting of detecting the instantaneous waterline from uprush and backwash using the Radon Transform, was introduced and validated using the the conventional color contrast method from RGB images and LiDAR measurements. The results show better detection skills even for adverse conditions.

### Witnessing African beaches changes

From the monitoring of topo-bathymetric variations in the storm-free environment of Grand Popo (Benin), beach profile evolution (shoreline and terrace, see Fig. 5) was observed to be dominated by seasonal to interannual scales^49,58^. The action of coastally-trapped waves (Kelvin waves) has also been demonstrated: a 7-cm change in sea level leads to nearly 2 m of horizontal terrace deformation^44^. Based on these video-derived morphological variations. Mingo et al.^59^ proposed and discussed a simple cubic parameterization based on the widely used Dean Number as the dominant control parameter for such beaches, while extending the common view of an equilibrium range profile as a power law of cross-shore distance.

The importance of intraseasonal to interannual variation of sea level on beach dynamics was also evidenced at James Town (Ghana). Angnuureng et al.^60^ demonstrated that shoreline changes are preferentially explained at daily scales by sea level anomaly (86%), waves (9%), and tide neap-spring cycles (5%) scales. These results show the role that the sea level anomaly can play in modulating the magnitude of wave action on the beach as stipulated by^44^.

### Revealing major drowning hazard

Rip currents usually occur at depth discontinuities in a sandbar, under a jetty or pier, which forces the discharging water into a thin corridor and accelerates the flow. These currents are one of the main hazards for beach users all over Africa and the world. And the studies carried out in Grand Popo (Benin) help to prevent and reduce the numerous cases of drowning deaths due to this phenomenon on the beaches of Africa.

Castelle et al.^61^ are the first to study the rip current system in West Africa using video images. Floc’h et al.^62^ developed a new method based on the colour processing of video images to detect the presence of rip currents via a change in turbidity. Morphological characteristics of the flash rip were computed from a set of instantaneous images and more than 50 rip events were counted per daylight, most occurring at low tide and migrating downdrift^62^.

Almar et al.^63^ described and tested a method based on the application of the Radon transform to directly estimate longshore currents using time-stack images. In addition, transient circulation was detected. Flash rips and surf eddies are transient horizontal structures of the order of 10 to 100 m, which can be generated in the surf zone in the absence of bathymetric irregularities. Using video-derived current fields, Marchesiello et al.^64,65^ revisited the processes of surf eddy generation with a new three-dimensional wave resolution model (CROCO) and provided a plausible demonstration of new 3D non-hydrostatic instability and turbulent cascade. Instant fields of surface current were retrieved from shore-based video cameras and unmanned aerial vehicles (*UAV*s) by an optical flow (OF) method named "*Typhoon*"^66^. Such computer vision algorithm estimates dense two-dimensional 2-component velocity fields from the observable motion of foam patterns in the surf zone.

## Coastal zone management

### Beach change monitoring

The video camera system installed at the enclosed Elmina beach (Ghana) is used, first, to test multi-platform monitoring of coastal erosion and secondly, to evaluate the effectiveness of beach defense structures. Two 200 m long jetties were built on either side of the mouth of Benya lagoon and a seawall was built at the fish-landing site near Elmina Castle, 800 m from the north side of the mouth of Benya Lagoon^67^. The multi-platform data collection strategy Angnuureng et al.^68,69^ was deployed for a year at Elmina (Ghana), through the use of the installed video camera system, drones (unmanned aerial vehicles), Sentinel satellite images, and a dumpy level (optical surveying levelling instrument). Angnuureng et al.^68^ revealed that data from local video cameras and drones are more effective for operational monitoring of shoreline changes at all-time scales compared to satellite imagery that is unsuitable for detecting daily or event-based beach changes needed by practitioners and management decisions. The obtained results indicated that the presence of the defense systems adequately protected the section of the beach, and a larger unprotected portion of the beach was out of balance with high erosion rates^68^. This is an example of the application of video data: to verify and evaluate the effectiveness of protective structures that are built along the coasts of Africa, and how they can induce morphological imbalances. This type of multi-platform monitoring allows coastal management to gauge the potential hazards associated with the building of houses and coastal infrastructure.

### Early warning system

The rapid development of video camera systems (see Fig. S2) now offers many applications that can provide quantitative information for swimmers, naval engineering, safety, and research, for predicting future hazards. A vulnerability evaluation of the coasts in Africa using data from video camera systems is feasible nowadays in a sustainable way, by defining risk indicators associated with the observations for providing early warning of potentially imminent hazards. The video camera systems could lead to the establishment of early warning systems for extreme events such as storms or floods on a regional scale. For example, in Senegal, the installed video camera can measure local conditions and serve as an alert system to fishermen in case of very dangerous swell conditions. In this case, the real-time processing of the data collected by the video camera system will facilitate the development of now-casting modules. In the locality of Cap Cameroon (Cameroon), which is inhabited mainly by fishermen, the recession of the coastline has led to the immersion of more than 300 hectares of coastal land per year^25^. Similarly, the Langue de Barbarie, near Saint Louis (Senegal), has undergone significant morphological changes following the opening of an artificial breach^70^.

The African coastal camera network offers the potential for more effective and controlled management of coastal development and protection. For example, the relocation and construction of houses on stilts can be better coordinated, rather than leaving them to the vagaries of weather and flooding. Indeed, the regular monitoring of oceanic forcing allows one to foresee with reasonable accuracy the associated morphological variations and thus to define zones at risk. The African coastal camera network represents an excellent risk assessment that could support an early warning tool for the security of people and goods and help with the security of infrastructures.

### Toward more science-based coastal zone management in Africa

According to^71^, coastal geophysical processes (particularly coastal erosion) and public policies are parts of the greatest challenges to be addressed and should be seen as a priority for research in Africa. They recommended an integrative conceptual framework that encompasses the study of phenomena ranging from geophysics to society, and the development of coastal management policies. Other studies (e.g.,^33,34^) have highlighted the interdependence between different coastal developments, whether along rivers or on the coast, on the overall sediment budget of the entire region, which is not limited to the geographic boundaries of countries. These studies recommended the development of a much more integrated large-scale coastal management plan. Unfortunately, the lack of timely, accurate, and trustworthy data has consistently been a problem in Africa. It still poses a significant obstacle to the efficient monitoring and assessment of development and intervention projects in the region, as data remain a critical tool for decision-making, development, and raw material for the quality of information.

The video camera network described in this paper could lead to the implementation of a more integrated management policy along the African coasts. Video camera systems can provide valuable information with adequate temporal resolution and spatial coverage^72^, serve as a multi-variable monitoring platform, and exhibit the ability to provide relatively continuous, nearshore real-time estimates (morphological and hydrodynamic) for periods stretching to decades on a spatial coverage of several kilometres, although more suitable for smaller-scale applications. Traditionally, in situ measurement techniques and other types of remote sensing (e.g., satellite, drone, Lidar) are either too costly or inadequate, and face logistical difficulties in deployment. In-situ techniques involve placing a vast array of instruments at a limited number of locations, lacking the spatial resolution and coverage required for effective coastal zone management. Satellite imagery has a low temporal resolution for detecting daily or event-based beach changes and low spatial resolution for coastal studies and management decisions. Video cameras provide metric or higher resolution at the coast for spatial coverage of a few kilometres^45^ and cover timescales from seconds to years and decades^68^. Moreover, the relatively low-cost of establishing a video camera system eases the spatial development of this network whose maintenance depends almost only on human resources. The multiplicity of video camera system sites contributes to the strengthening of the database necessary to study the associated coastal physical phenomena.

The database generated from the African coastal camera network should serve as a basis for the study of human and natural processes (e.g., climate change), the prediction of associated risks, and therefore for the development of a common regional coastal management policy. This includes the identification of objectives, priorities, strategies, and timetables common to several countries to achieve them, even if compliance with legal and regulatory frameworks is a challenge that falls under the sovereignty of the States, with of course the consideration of United Nations guidelines at the level of the African Union. Thus, the ongoing data acquisition by video cameras could be a crucial backbone for coastal monitoring and management along the African coast.

The African coastal camera network can be the basis for the development of monitoring systems specific to the sub-region concerning the management of, for example, the registration of the departure and arrival of artisanal fishing boats, which are essential in the region. More rigorous observations using video stations would likely provide a better understanding of the local dynamics of the Langue de Barbarie (Senegal), which continues to undergo significant morphological changes following the opening of an artificial breach^70^. Algorithms to detect plastic waste or oil pollution from video-derived images in the nearshore could be developed and tested, for example in coastal areas where oil facilities are located. Monitoring of the sediment budget due to coastal infrastructure development is feasible using a similar approach to that used in the multi-platform data collection strategy deployed for a year at Elmina, Ghana^68,69^. Other local challenges, such as Sargassum beach landings^73,74^ are becoming an increasingly prevalent problem in the West African region (e.g. Ghana). Innovative video image processing algorithms could provide evidence of the spread of Sargassum in the nearshore.

The African coastal camera network has the potential to bridge the problem of non-data availability for coastal studies in Africa. The video data from the network can provide data and information that will help coastal scientists, investors, managers, and policymakers in improving the spatial planning and management of the African coastal areas. The network can be used to gather data that will be used for studies on flooding, erosion, coastal structures, infrastructure monitoring, oil pollution assessment, overfishing, and ecosystem and ecosystem services. Such a network should be linked to the World Bank-funded *WACA* Management Programme; or the *ORLOA*, an observatory established to develop the Central and West African coastal territories sustainably, through the promotion, production, and sharing of reliable and homogeneous data using harmonized data collection protocols for decision-making.

## Future perspective

### Technological improvements

A video camera system consists of a hardware part (camera and/or local computer) and a software part (processing algorithms). Although video camera systems do not experience issues associated with in situ instrumentation (e.g., flow disruption, biofouling, and sensor degradation under unfavourable wave conditions), they are still subject to other types of malfunctions: air moisture very often causes electronic failures of the station and power failures often lead to a more or less prolonged downtime of the camera. In addition, video camera systems are limited to daytime observations and atmospheric conditions can affect image quality (dew or rain on the lens of the camera, fog, and sunlight).

To reduce the effects of air moisture, cameras fitted with memory cards have been installed in Saint Louis (Senegal), Assouindé (Côte d’Ivoire), Dzita (Ghana), and Elmina (Ghana), instead of installing an on-site local computer as in Grand Popo (Benin), Mbour (Senegal), Kribi (Cameroon) and James Town (Ghana). In Saint Louis (Senegal), the video camera system is equipped with solar panels to compensate for power failures. The use of solar panels reduces gaps in data collection. The implementation of remote control system which is feasible when an internet connection is available would allow real-time monitoring essential for early warning systems. The effects of atmospheric conditions on image quality are compensated by applying image quality detection algorithms, such as the criterion based on the standard deviation of the pixel intensity^45,75^.

### African coastal camera network management

The African coastal camera network is rapidly growing, and it is set to become an African data hub for coastal video imagery. The *ORLOA* could be used as a regional centralized repository accessible to researchers and decision makers. The repository should be made up of consistently data and processing algorithms, to promote sharing and integration. The network is poised to make significant progress on coastal environmental issues that have significant societal impacts. It is, therefore, essential to expand the video camera station coverage to other locations within Africa, especially the coastal sectors that have been characterized and identified based on their level of hazards and issues, or that are characterized by varying morphology and dynamics.

In the long-term, the video-derived data has considerable potential for use in other coastal environmental issues such as in the management of ecosystem protection, including competing coastal land uses (e.g., conservation vs. development), fisheries, and resource management, recreation, plastic and oil pollution monitoring, navigation, and coastal protection. Similarly, it has the potential of providing key information that can be used in basic input to the engineering design of the coastal zone, and support for integrated numerical model validation. And video-derived data can be coupled with other available data/platforms to provide even more observations. This should be strongly encouraged. However, strengthening the existing video camera systems and creating new ones requires more stable funding. The African and international coastal community, including academics, governments, regional (*WACA*/*ORLOA*), and local and international development partners are welcome to be part of the network. If this vision is achieved, coastal video camera imagery will transform environmental monitoring along the African coasts. It is the only way forward if we want sustainable African coasts.

## Conclusions

This paper presents a pilot active network of video camera systems along the African coasts with similar technology, methods, and data sharing between active scientific communities in these countries.

- This network fulfills a need to document African coastal evolution with the objectives of creating an efficient coastal database, implementing targeted research, promoting a more effective land-use planning policy and coastal zone management.
- The database consists of hydrodynamic (coastal waves, tides, and sea level) and morphologic (shoreline, beach profile and bathymetry) data collected by an increasing number of video camera systems. These data have led to new methodological developments, student capacity building, and a better understanding of coastal processes, e.g., tropical beach dynamics, sea level variability, dangerous rip currents or flash rips, and the impact of coastal defences.
- The African coastal camera network can help investigate and address societal and environmental challenges (e.g., engineering, risk mitigation, and prevention). It can also provide key local ground truth that is important for satellite remote sensing techniques and engineering studies, even more, if when coupled with other measurements such as *DGPS*, unmanned aerial vehicles (*UAV*s), but also wave, tide and current moorings.
- The development efforts and lessons learned in each African country can be synergized, enabling more efficient planning through sharing of associated (novel) technologies, data analyses, and results. This network is a real asset for regional programs like the West African Coastal Observatory (*ORLOA*) or the West Africa Coastal Areas Program (*WACA*) to target hotspots. This network needs to be perennialized and further extended to answer important questions related to coastal hazard hotspots and coastal zone development in Africa. The expansion of the video station network and additional efforts from all coastal planners and development stakeholders are required.
- The low-cost of establishing a video camera system is an advantage that will facilitate its replicability and the expansion of this observation network.

## Data and methods

**Figure 7:**
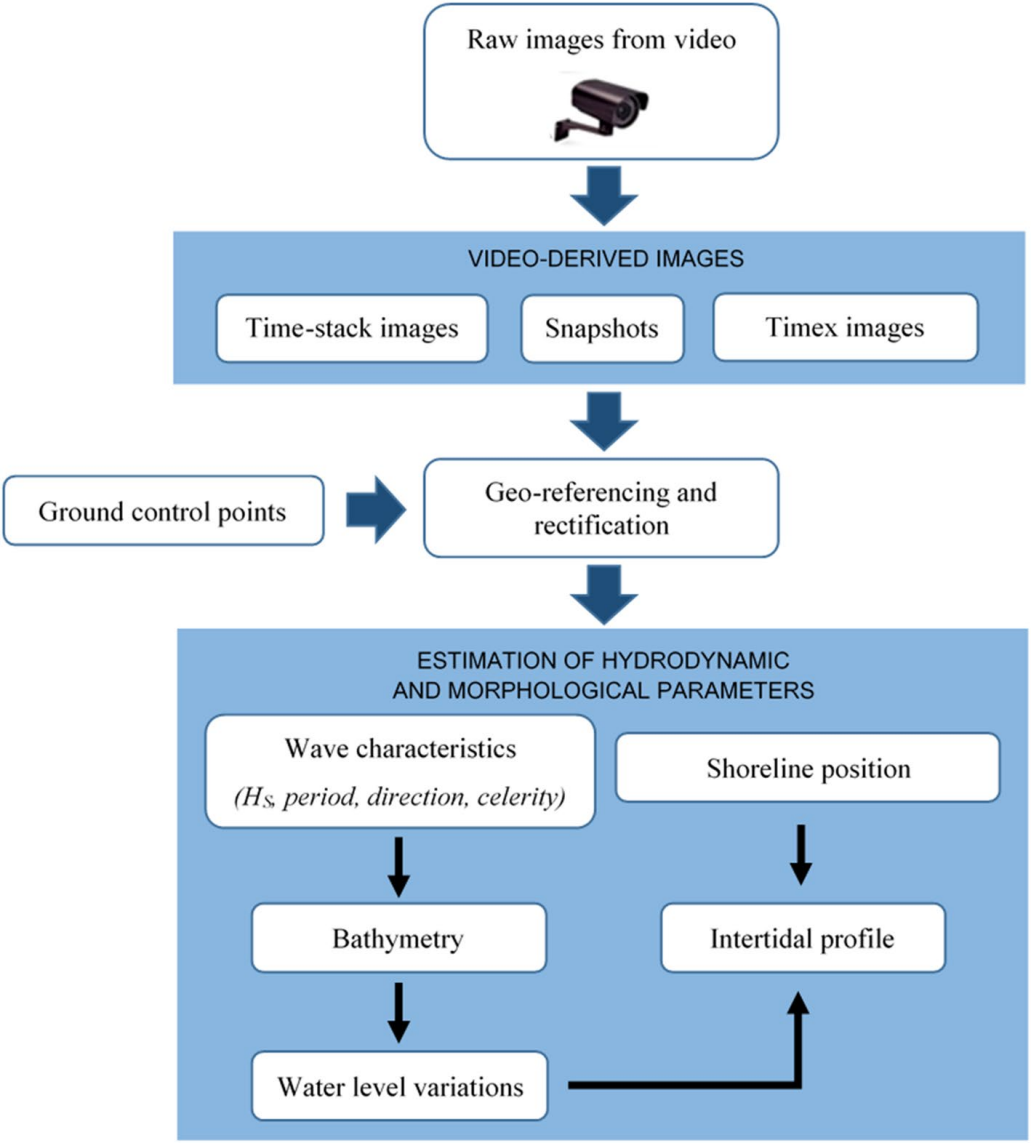
Full video monitoring process: from data collection to post-processing.

Several hydrodynamic and morphological parameters can be deduced from video imagery. Hydrodynamic parameters consist of wave characteristics (${H}_{s}$, ${T}_{p}$, direction) and water level variations (tide and sea level rise). Morphological parameters consist of shoreline location, intertidal topography, and bathymetry. Figure 7 shows the complete processing scheme and the estimated parameters.

### On site-processing

Grand Popo (Benin), James Town (Ghana), Mbour (Senegal), and Kribi (Cameroon) video camera systems are fitted with an on-site local computer that processes the raw images and stores three types of secondary images every 10 or 15 min: snapshots, time exposures (timex), and time-stacks. Timex images are snapshot images averaged every 10 or 15 min into a single image^76^. Time-stack images are intensity values saved at each time step at a selected array of pixels from snapshots^77^.

Saint Louis (Senegal), Elmina (Ghana), Dzita (Ghana) and Assouindé (Côte d’Ivoire) video camera systems are not fitted with a local computer. The raw images collected at a frequency of 2 Hz are stored directly on a memory card as a video file since the new versions of the cameras used allow this. This implies that the data must be retrieved before the memory card is full.

### Geo-referencing and image rectification

The process of image rectification consists of determining the relationship between the original image pixels coordinates and the real world coordinates: latitude, longitude and elevation (see Fig. S1 for the case of Grand Popo, Benin). The process is accomplished by direct linear transformation using the ground control point’s coordinates^76^ and radial distortion correction of the lens^78^. The image distortion induced by the curve of the lens is corrected using the internal parameters of the camera (focal length and distortion coefficients) which are calculated using the least squares error minimization method.

Overall, the average horizontal error due to image rectification varies from a few centimetres on the beach to a few metres offshore^45^. However^79^, showed that the local video station settings (camera height and pointing angle) add errors of a few metres to rectification error, in addition to uncertainties related to camera movements due to atmospheric conditions^80^.

### Post-processing

Table 1 summarizes the complete list of parameters estimated from the images collected by the African coastal camera network and related references in the literature.

**Table 1:** Reported methods for processing video images and estimating coastal hydrodynamic and morphological parameters.

Reported video-derived parameters		Source	Automatic detection method	References
Hydrodynamic parameters	${H}_{S}$	Time-stacks	Breaking wave height direct estimator	^81^
	Wave period	Time-stacks	Upward zero-crossing method	^48^
	Wave direction	Timex and snapshots	Subtracting the timex image from the instantaneous snapshot to remove the bottom (which does not move) and bring out the wave crests (which do move)	^48,82^
	Wave celerity	Pixel intensity from snapshots	Spectral approach: cBathy	^42,43,83^
		Time-stacks	Temporal approach: inter-correlation	^43,45,46,52^
	Water level variations	Bathymetry	Removing the moving average of instantaneous depths for one or more tidal cycles	^45,46^
	Longshore currents	Time-stacks	Radon transform	^63^
Morphological parameters	Shoreline position	Timex	Minimum Shoreline Variability (MSV)	^39^
	Intertidal profile	Timex and water level variations	Delineation of the shoreline at different tidal levels, interpolated between low and high tides day by day	^48^
	Bathymetry	Wave celerity	Celerity-based depth inversion method using the linear dispersion relation for surface free waves	^42,43,52,83^

## Supplementary Information

Supplementary Information.

## Data Availability

The datasets generated by the African coastal camera network are available from the authors on reasonable request. Correspondence and requests should be addressed to G.O.A and R.A. The processing codes used are available on https://github.com/gregsolo/CoastCAMs.git.
