# Supplementary material for: African coastal camera network efforts at monitoring ocean, climate, and human impacts
Source: Sci Rep. 2023 Jan 27;13:1514. doi: 10.1038/s41598-023-28815-6 (PMC9883390; doi:10.1038/s41598-023-28815-6)
Supplement: Supplementary file 1 — Supplementary Information. [file 41598_2023_28815_MOESM1_ESM.docx]

**SUPPLEMENTARY MATERIAL for**

**African Coastal Camera Network efforts at monitoring Ocean, Climate, and Human Impacts**

G. O. Abessolo^1*^, D. B. Angnuureng^2^, R. Almar^3^, F. Bonou^4,5^, Z. Sohou^6^, I. Camara^7^, A. Diouf^8^, G. Alory^3^, R. Onguéné^9^, A. C. Mama^1^, C. O. T. Cissé^10^, B. A. Sy^10^, I. Sakho^11,12^, S. Djakouré^13^, S. Yao^14^, A. R. Tano^15^, E. W.J. Bergsma^16^, O. Dada^3,17^

^1^ Ecosystems and Fishery Resources Laboratory, Institute of Fisheries and Aquatic Sciences, University of Douala, BP 2701 Douala, Cameroon

^2^ Africa Centre of Excellence in Coastal Resilience, CCM, University of Cape Coast, Ghana

^3^ LEGOS, OMP, UMR 5566 (CNES-CNRS-IRD-University of Toulouse), Toulouse, France

^4^ Laboratoire d’Hydrologie Marine et Côtière, Institut de Recherches Halieutiques et Océanologiques du Bénin (IRHOB)/ Laboratoire de Physiques et Applications, LPA/ Université Nationale Des Sciences Technologies, Ingénierie Et Mathématiques (UNSTIM)

^5^ Chaire Internationale de Physique, Mathématiques et Applications

^6^ Laboratoire d’Hydrologie Marine et Côtière, Institut de Recherche Halieutique et Océanologique du Benin (LHMC-IRHOB), Cotonou, Benin.

^7^ Cheikh Anta Diop University, Dakar, Senegal

^8^ Laboratoire d’Océanographie, des Sciences de l’Environnement et de Climat (LOSEC), Université Assane Seck, BP 523, Ziguinchor, Sénégal

^9^ Technology and Applied Science Laboratory, University Institute of Technology, University of Douala, Douala P.O. Box 8698, Cameroon

^10^ University Gaston Berger, Laboratory Leïdi "Dynamics of the territories and development", BP 234 Saint-Louis, Senegal

^11^ Université Amadou Mahtar Mbow de Dakar à Diamnadio, UMR Sciences, Technologies Avancées et Développement Durable, BP 45927. Dakar – Senegal;

^12^ Univ Rouen Normandie, UNICAEN, CNRS, M2C UMR 6143, F-76000 Rouen, France

^13^ Laboratoire des Sciences de la Matière, de l'Environnement et de l'Energie Solaire (LASMES)/UFR SSMT/Université Félix Houphouët-Boigny, Cocody, Côte d'Ivoire

^14^ Laboratoire de Physique et de Géologie Marine, Centre de Recherches Océanologiques –Abidjan / Côte d’Ivoire

^15^ Laboratoire de Physique Fondamentale et Appliquée, Université NANGUI ABROGOUA, Abidjan, Côte d'Ivoire

^16^ CNES, 18 Avenue Edouard Bélin, 31400 Toulouse, France

^17^ Department of Marine Science and Technology, Federal University of Technology, Akure, Nigeria

^*^ Corresponding author: [gregoireabessolo@ish.cm](mailto:gregoireabessolo@ish.cm) / [gregsolo55@yahoo.fr](mailto:gregsolo55@yahoo.fr)

Table S1: Technical, morphological, and hydrodynamic characteristics of video camera system stations in Africa. The *RTR* parameter represents the Relative Tidal Range (see [1]). The morphological data of the beaches were collected during field measurement campaigns (*e.g.*, [2-6]). The characteristics of waves were derived from European Re-Analysis ERA5 datasets. ERA5 waves were propagated to the shore using the formula by [7]. ERA 5 data are freely available at [www.ecmwf.int/en/forecasts/datasets/reanalysis-datasets/era5](http://www.ecmwf.int/en/forecasts/datasets/reanalysis-datasets/era5). Tidal data were collected from the available literature review.

| *Video camera station* | | *1* | | *2* | *3* | *4* | *5* | *6* | *7* | *8* |
| --- | --- | --- | --- | --- | --- | --- | --- | --- | --- | --- |
| *Site* | *Country* | *Benin* | | *Ghana* | *Senegal* | *Cameroon* | *Ghana* | *Ghana* | *Senegal* | *Côte d’Ivoire* |
|  | *Town* | *Grand Popo* | | *James Town* | *Mbour* | *Kribi* | *Dzita* | *Elmina* | *Saint Louis* | *Assounidé* |
|  | *Location* | 6.2°N  1.7°E | | 5.5°N  0.2°W | 14.4°N  16.9°W | 2.9°N  9.9°E | 5.7°N  0.8°W | 5.1°N  1.3°W | 16.0°N  16.5°W | 5.2°N 3.5°W |
| *Setup* | *Number of cameras* | 2 | | 1 | 1 | 1 | 1 | 1 | 1 | 1 |
|  | *Activity period (until 2022/08)* | 2013/02 to  2021/03 | 2018/07  to  2021/03 | 2013/08  to  2015/02 | 2014/12  to  2016/12 | 2015/05  to  2018/03 | 2018/04  to  2020/12 | 2018/11  to  2022/01 | 2021/01  to  2022/08 | 2022/07  to  2022/08 |
|  | *Resolution (pixels)* | $1600\times728$ | $1920\times1080$ | $1280\times728$ | $1176\times720$ | $1600\times1200$ | $7552\times1416$ | $1600\times720$ | $7552\times1416$ | $7552\times1416$ |
|  | *Elevation (m)* | 20 | | 40 | 6 | 8 | 13 | 40 | 20 | 11.5 |
|  | *Horizontal viewing angle (°)* | 93 | 93 | 71.2 | 93 | 71.2 | 180 | 180 | 180 | 180 |
|  | *Distance to water limit (m)* | 70 | | 90 | 45 | 15 | 5 | 80 | 100 | 50 |
|  | *Activity (local time)* | 7:00 to  18:00 | | 7:00 to 18:00 | 7:00 to 18:00 | 7:00 to 18:00 | 7:00 to18:00 | 7:00 to 18:00 | 10:00 to 11:00 | 10:00 to 13:00 |
|  | *Framerate (Hz)* | 2 | | 2 | 2 | 2 | 2 | 2 | 2 | 2 |
|  | *Data storage* | On-site  computer | | On-site computer | On-site computer | On-site computer | Memory card | Memory card | Memory card | Memory card |
| *Beach* | *Type* | Open | | Open | Open | Open | Open | Enclosed | Open | Open |
|  | *Intertidal slope (rad)* | 0.08 | | 0.03 | 0.05 | 0.03 | 0.1 | 0.08 | 0.05 | 0.03 |
|  | *D_50_ (mm)* | 0.6 | | 0.6 | 0.4 | 0.5 | 0.8 | 1 | 0.3 | 0.6 |
| *Waves* | *H_S_ (m)* | 1.4 | | 1.4 | 1.5 | 0.9 | 1.3 | 1.2 | 1.5 | 1.2 |
|  | *T_p_ (s)* | 9.4 | | 10.9 | 9.2 | 11 | 10.9 | 10.9 | 9.2 | 9.1 |
|  | *Direction* | S-SW | | S-SW | NW-SW | SW | S-SW | S-SW | NW | S-SW |
| *RTR* | | 1 | | 1 | 1.1 | 1 | 1.1 | 1 | 1.1 | 1.2 |

Table S2: Correlations *r*, root mean square (*RMS*) differences and mean errors (*ME*) synthesis of video-derived morphological and hydrodynamic parameters with Grand Popo (Benin) 2014 field measurements [2], *CTOH X-TRACK* and *SSALTO/DUACS* altimetry products, European Re-Analysis ERA5 data, and WaveWatch III model outputs. WaveWatch III and ERA-Interim waves were propagated to the shore using the formula by [7]. Grand Popo (Benin) 2014 field measurements consist of wave characteristics and tide collected with an Acoustic Doppler Current Profiler (ADCP) moored at 10-m depth, beach topography collected with a Differential Global Positioning System (DGPS), and bathymetry collected with a bathymetric sonar (From [8-12]).

| Video-derived parameters | Field data  (March 11-19, 2014 ) | SSALTO/DUACS  (February 2013 - August 2016) | CTOH-XTRACK  (February 2013 - August 2016) | WaveWatch III  (February 2013 - August 2016) |
| --- | --- | --- | --- | --- |
| $H_{s}$ (m) | *RMS*: 0.14  *ME*: - 0.02 | // | // | *r*: 0.89  *RMS*: 0.3  *ME*: 0.3 |
| $T_{m}$ (s) | *RMS*: 1.31  *ME*: - 0.18 | // | // | *r*: 0.66  *RMS*: 2.4  *ME*: 2.3 |
| Wave direction (°) | *RMS*: 9.25  *ME*: 2.25 | // | // | *r*: 0.44  *RMS*: 9.4  *ME*: 8.5 |
| Intertidal topography (m) | *RMS*: 0.28  *ME*: 0.23 | // | // | // |
| Bathymetry (m) | *RMS*: 1.62  *ME*: 0.27 | // | // | // |
| Water level variations (m) | *r*: 0.90  *RMS*: 0.20  *ME*: 0.02 | *r*: 0.58  *RMS*: 0.05 | *r*: 0.56  *RMS*: 0.06 | // |


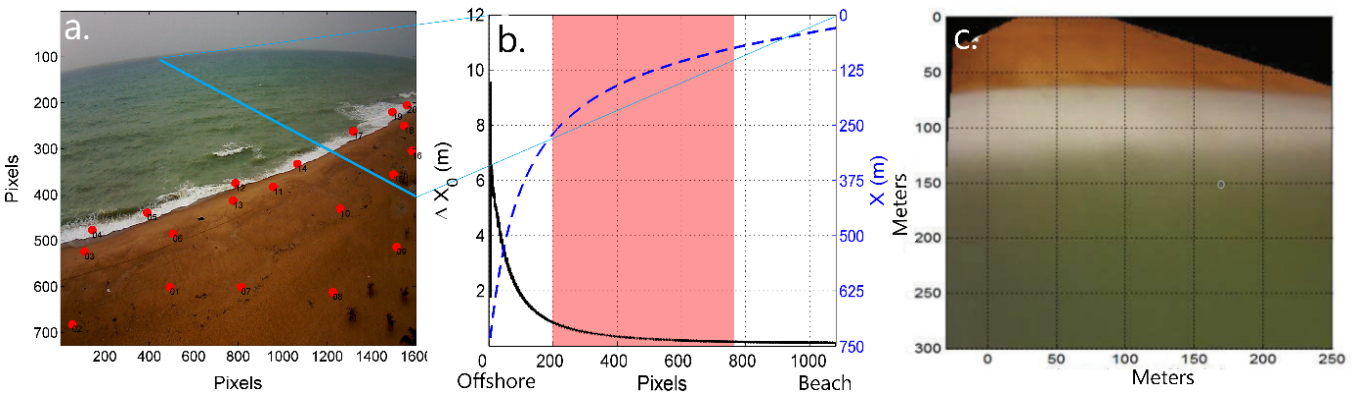


Figure S1: Image rectification at Grand Popo, Benin: (a) Grounds control points in red on a snapshot derived from the video camera. The blue line indicates the cross-shore time-stack location. (b) Averaged pixel footprint ∆X_0_ and cross-shore coordinate X in meters relative to the camera location, versus pixel coordinate on the cross-shore stack. The shaded area in red indicates the considered area, which corresponds to the submerged part of the stack with a pixel footprint of less than 1 m. (c) Rectified timex showing the camera field of view.


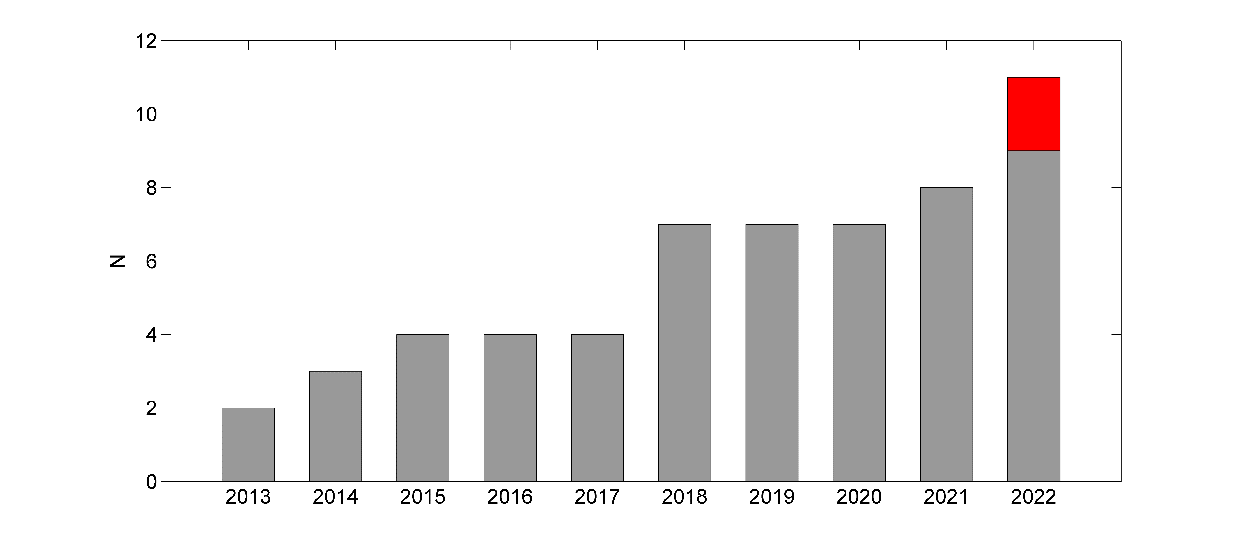


Figure S2: Cumulative number of cameras (N) installed along the African coast per year. The section in red represents the cameras that are being installed in 2022 and are not yet active.

**References**

[1] Masselink, G. & Short, A.D. The effect of tidal range on beach morphodynamics and morphology: a conceptual beach model. *Journal of Coastal research*, **9(3),** 785-800 (1993).

[2] Almar, R. *et al*. The Grand Popo beach 2013 experiment, Benin, West Africa: from short timescale processes to their integrated impact over long-term coastal evolution. *Journal of Coastal Research*, **70,** 651–656, (2014).

[3] Ndour, A. *et al*. On the natural and anthropogenic drivers of the Senegalese (West Africa) low coast evolution: Saint Louis beach 2016 COASTVAR experiment and 3D modeling of short term coastal protection measures. In: Malvárez, G. and Navas, F. (eds.), Global Coastal Issues of 2020, *Journal of Coastal Research*, Special Issue **95,** 583-587 (2020).

[4] Angnuureng, D. B., Addo, K. A., Almar, R. & Dieng, H. Influence of sea level variability on a micro-tidal beach. *Nat Hazards*, **68,** <https://doi.org/10.1007/s11069-018-3370-4> (2018).

[5] Angnuureng, D.B. *et al*. Application of shore-based video and unmanned aerial vehicles (drones): complementary tools for beach studies. *Remote Sensing*, **12,** 394–413 (2020).

[6] Yao, K.S., Dangui, N.P., Gbegbe M. & Abe, J. Caractéristiques du climat de houle au large de la côte ivoirienne entre 1985 et 2015 : impacts sur l'évolution morpho dynamique du rivage. *Actes du colloque international XV èmes Journées Nationales Génie Côtier – Génie Civil*, 29 - 31 mai 2018, La Rochelle, France, pp. 313-320, <https://doi.org/10.5150/jngcgc.2018.035> (2018).

[7] Larson, M., Hoan, L. X. & Hanson, H. A direct formula to compute wave properties at incipient breaking. *Journal of Waterway, Port, Coastal and Ocean Engineering*, **136(2),** 119–122 (2010).

[8] Abessolo, O. G. *et al*. Potential of video cameras in assessing event and seasonal shoreline behaviour: A case study at Grand Popo, Benin (Gulf of Guinea). *Journal of Coastal Research*, SI **75,** 442–446 (2016).

[9] Abessolo, O. G. *et al*. Beach response to wave forcing from event to inter-annual time scales at Grand Popo, Benin (Gulf of Guinea). *Water*, **9,** 447; <https://doi.org/10.3390/w9060447> (2017).

[10] Abessolo, G.O. *et al*.. Sea level at the coast from video-sensed waves: comparison to tidal gauges and satellite altimetry. *Journal of Atmospheric and Oceanic Technology*, **36,** 1591-1603 (2019).

[11] Abessolo, O. G., Almar, R., Bonou, F. & Bergsma, E. J. Error proxies in video-based depth inversion: temporal celerity estimation. In: Malvárez, G. and Navas, F. (eds.). Global Coastal Issues of 2020, *Journal of Coastal Research*, Special Issue **95,** 1101–1105 (2020).

[12] Abessolo, G.O., Almar, R., Jouanno, J., Bonou, F., Castelle, B. & Larson, M. Beach adaptation to intraseasonal sea level changes. *Environmental Research Communications*, **2,** 051003; <https://doi.org/10.1088/2515-7620/ab8705> (2020).
